# EMMA 2 – A MAGE-compliant system for the collaborative analysis and integration of microarray data

**DOI:** 10.1186/1471-2105-10-50

**Published:** 2009-02-06

**Authors:** Michael Dondrup, Stefan P Albaum, Thasso Griebel, Kolja Henckel, Sebastian Jünemann, Tim Kahlke, Christiane K Kleindt, Helge Küster, Burkhard Linke, Dominik Mertens, Virginie Mittard-Runte, Heiko Neuweger, Kai J Runte, Andreas Tauch, Felix Tille, Alfred Pühler, Alexander Goesmann

**Affiliations:** 1 Computational Genomics, Center for Biotechnology, Bielefeld University, 33594 Bielefeld, Germany; 2 Lehrstuhl für Bioinformatik, Friedrich-Schiller-Universität Jena, 07743 Jena, Germany; 3 Institute for Medical Biology, Department of Molecular Biotechnology, Medical Faculty, University of Tromsø, 9037 Tromsø, Norway; 4 Institute for Genome Research and Systems Biology, Bielefeld University, Bielefeld, Germany; 5 International NRW Graduate School in Bioinformatics and Genome Research, Center for Biotechnology, Bielefeld University, Bielefeld, Germany; 6 Fakultät für Biologie, Bielefeld University, Bielefeld, Germany; 7 Institute for Plant Genetics, Unit IV – Plant Genomics, Leibniz Universität Hannover, 30419 Hannover, Germany

## Abstract

**Background:**

Understanding transcriptional regulation by genome-wide microarray studies can contribute to unravel complex relationships between genes. Attempts to standardize the annotation of microarray data include the Minimum Information About a Microarray Experiment (MIAME) recommendations, the MAGE-ML format for data interchange, and the use of controlled vocabularies or ontologies. The existing software systems for microarray data analysis implement the mentioned standards only partially and are often hard to use and extend. Integration of genomic annotation data and other sources of external knowledge using open standards is therefore a key requirement for future integrated analysis systems.

**Results:**

The EMMA 2 software has been designed to resolve shortcomings with respect to full MAGE-ML and ontology support and makes use of modern data integration techniques. We present a software system that features comprehensive data analysis functions for spotted arrays, and for the most common synthesized oligo arrays such as Agilent, Affymetrix and NimbleGen. The system is based on the full MAGE object model. Analysis functionality is based on R and Bioconductor packages and can make use of a compute cluster for distributed services.

**Conclusion:**

Our model-driven approach for automatically implementing a full MAGE object model provides high flexibility and compatibility. Data integration via SOAP-based web-services is advantageous in a distributed client-server environment as the collaborative analysis of microarray data is gaining more and more relevance in international research consortia. The adequacy of the EMMA 2 software design and implementation has been proven by its application in many distributed functional genomics projects. Its scalability makes the current architecture suited for extensions towards future transcriptomics methods based on high-throughput sequencing approaches which have much higher computational requirements than microarrays.

## Background

The introduction of microarray technology has marked a paradigm shift in genomics. The first studies in 1995 [1,2] showed its potential for the functional analysis of genomes. Instead of being focused on single or small sets of genes of interest the microarray technology provides a holistic view on gene expression of an organism due to the analysis of thousands of genes in a single experiment. This high-throughput technique is used in functional genomics due to its wide range of applications, relative cost-efficiency, and its potential to support genome-wide studies [3].

Practices in functional genomics have reached a turning point where standardization of data storage, management and data-mining of large data sets, documentation of experimental processes, and a guidance on the applicable analysis methods have gained an increasing importance [4]. Microarray experiments are often conducted within larger international projects with a large number of participants. To standardize the annotation of microarray data, the Minimum Information About a Microarray Experiment (MIAME) [5] recommendation, the Microarray Gene Expression Markup Language (MAGE-ML) format for data interchange [6], and ontologies such as the MGED ontology [7] have been proposed.

There exists a variety of software applications aiming to simplify the analysis, annotation, storage and retrieval of experiments and data. Also public repositories such as ArrayExpress [8] and GEO [9] have been developed to integrate data analysis tools and gene expression visualization.

As has been recently noted combining gene expression data with functional annotation data such as metabolic pathways, gene regulatory networks or metabolome data can open new perspectives for generating hypotheses and understanding regulatory interactions [10,11]. The substantial benefits of data integration are the availability of up-to-date functional knowledge from external or internal sources and taking advantage of external data within new data analysis methods.

As an example for an approach to integrate data the Genome Expression Pathway Analysis Tool (GEPAT) offers an analysis of gene expression data under genomic, proteomic and metabolic context. It supports various normalization and analysis methods, but it does not support MAGE-ML, the MGED ontology, and web-services [12].

The MARS system is an example for database software that provides MAGE-ML support and web-service access to raw data [13]. It provides no integrated analysis functionality, instead it requires external software which requires to transfer large files between applications. BASE 2 is another database application that supports MAGE-ML export and has an integrated analysis system using pipelines [14]. Furthermore, it can make use of the CARMAweb [15] web-service for normalization of spotted and Affymetrix arrays. Currently BASE 2 does not support MAGE-ML import and integrated cluster analysis. Although the MAGE-ML format is a widely accepted standard, none of the mentioned microarray data analysis tools support import and export of the complete model, and only loosely integrate the MGED-ontology into the annotation of experimental conditions. It is also desirable to allow queries for gene expression data over system-independent interfaces such as SOAP-based web-services.

To address these requirements, a novel standards-compliant system, EMMA 2, was developed based on the experiences gained with its predecessor EMMA 1 [16]. EMMA 2 has been completely implemented from scratch as EMMA 1 was based on a self-designed persistent data model which imposed large limitations with respect to full MAGE-OM support. Also, the EMMA 1 system did not support data integration via web-services. The data model also restricted the application of EMMA 1 to dual channel arrays because the aspect of two channels was strongly represented in the design of the system. While EMMA 1 is capable of handling different normalization and test methods, it did not support to keep more than one result within one experiment at a time, which would be important for method comparison.

All steps in the workflow of an experiment are annotated in a MAGE-compliant way, ranging from acquisition of experimental protocols to handling of raw and transformed data. The system also provides a close integration of other data-sources, such as genome annotations, metabolic pathways, and proteome measurements. In contrast to other microarray databases and analysis tools, EMMA 2 provides a wide collection of algorithms and a database to store, retrieve, and comprehensively analyze genome-wide datasets in a MIAME and MAGE-ML compliant format.

## Implementation

### Architecture

The application is realized using an extended 3-tier architecture. The standard 3-tier approach was augmented by using the object-relational mapping layer O2DBI, which provides a fully object-oriented abstraction to the underlying database. O2DBI uses MySQL tables to store persistent Perl objects and can convert data models into SQL and Perl code automatically.

**Figure 1:**
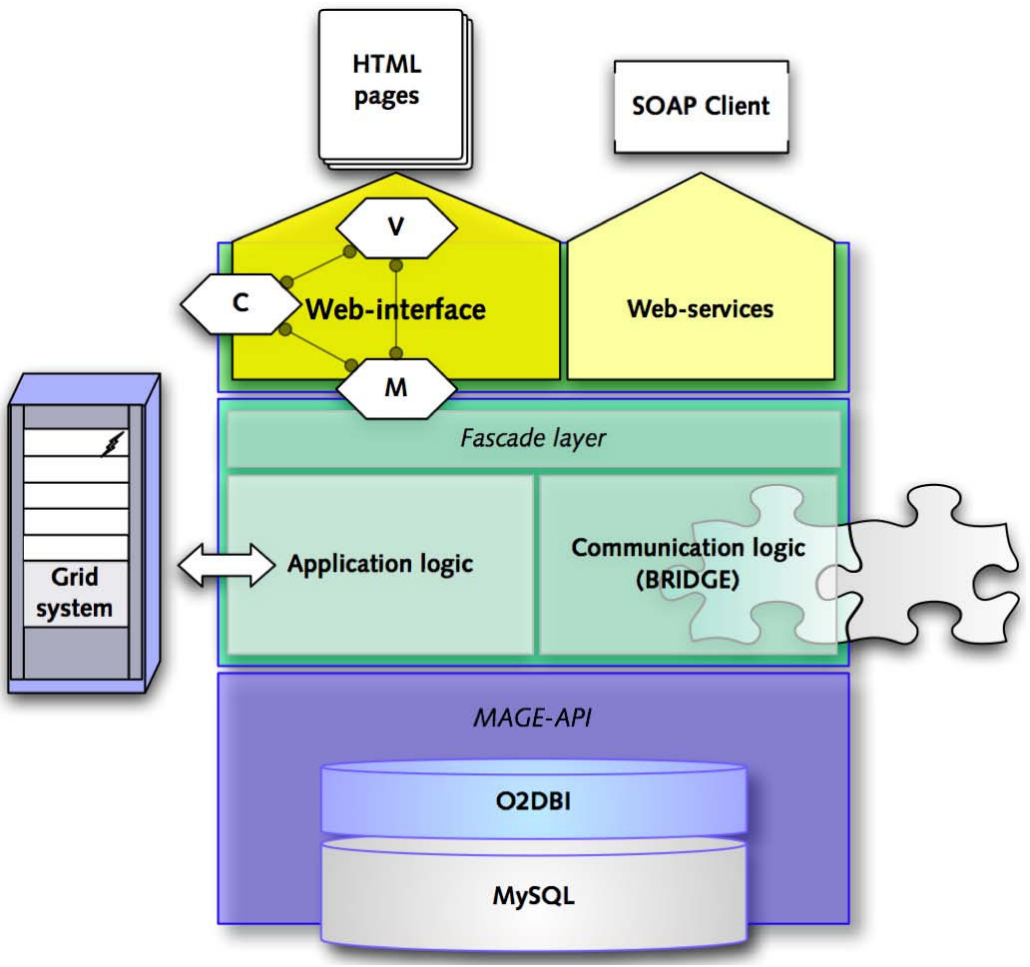
**Architecture of the EMMA 2 software**. EMMA 2 is built by combining a three-tier architecture in combination with a model-view-controller (MVC) design pattern. In contrast to the standard three-tier approach, the back-end-layer (bottom) contains an object-relational mapping layer (O2DBI). The business-layer contains the application logic and provides data integration features. The presentation layer provides two modes of presenting data.

Furthermore, we have added an additional communication layer to the application tier (BRIDGE), which is responsible for the communication with remote applications [11]. Finally, the presentation layer is divided into the web-interface and the web-services interface that provides SOAP based web-services. Within the web-interface layer, we have adapted the concept of the Model-View-Controller (MVC) design pattern (see Figure 1) [17].

### Model Driven Approach (MDA)

The system was designed and implemented using a model driven approach (MDA) [18]. Within the MDA framework, object-oriented methods were applied during the design process using the Unified Modelling Language (UML).

We decided to implement the full MAGE object model (MAGE-OM) as the core database model, because we wanted to provide full support for each valid MAGE-ML document. This is made possible by providing a one-to-one implementation of the classes found in MAGE-OM within our framework. As MAGE-OM is very complex and in order to speed up implementation of the database layer and the application API, we decided to automate the code-generation process of the application logic, database back-end, and MAGE-ML export classes.

Two auxiliary model components have been added to supplement MAGE-OM. The access control model (ACL-OM) implements access control lists to be able to configure access to each MAGE-OM object. The second addition contains classes for the execution of analysis tools and the storage of results (TOOLS-OM). Both models are only sparsely interlinked with MAGE-OM, which makes them re-usable for other applications.

To increase the efficiency of the database we have optimized the automatically generated SQL model. The optimizations include additional SQL indices and SQL views for frequently used queries such as the mapping between features, reporters, and composite sequences. The mapping between these data structures is highly complex in MAGE-OM as it involves join operations over multiple tables and can be simplified using SQL views using one virtual table.

### Development Tools and Dependencies

The system is implemented using Perl, the Apache web-server, and MySQL databases as storage back-end. For the purpose of code-generation from XML definitions we used XSL transformations (XSLT).

The web-interface is based on an Apache web-server, the CGI Perl module, HTML templates and Ajax to improve interactivity. To browse cluster analyses, the web-interface contains a Java-applet. We use the SOAP::Lite Perl module for client-server communication via web-services.

Analysis functions are implemented using the R statistical programming environment [19] and Bioconductor packages [20]. We are using the RSPerl module from the Omegahat project to embed R within our Perl application [21]. This module allows to flexibly create and transfer data-structures between both languages and avoids data-exchange by passing temporary files.

Microarray data sets consist mainly of large numeric data arrays of variable length dimensions. Relational database tables are not the optimal storage engine for this type of data. Therefore, numeric quantification data are stored efficiently and in a platform-independent way using HDF5 (Hierarchical Data Format) [22] files. We use the Perl Data Language (PDL) as an efficient interface to HDF5 files.

### Supported Formats

The system supports the upload of array data using the Imagene or Genepix software formats as well as general comma or tab-separated files and CEL-files. For NimbleGen arrays, pair files containing pairwise probe intensities are imported. Due to the modular architecture of the importer, new formats can be added easily.

## Results

### The Web-Interface

**Figure 2:**
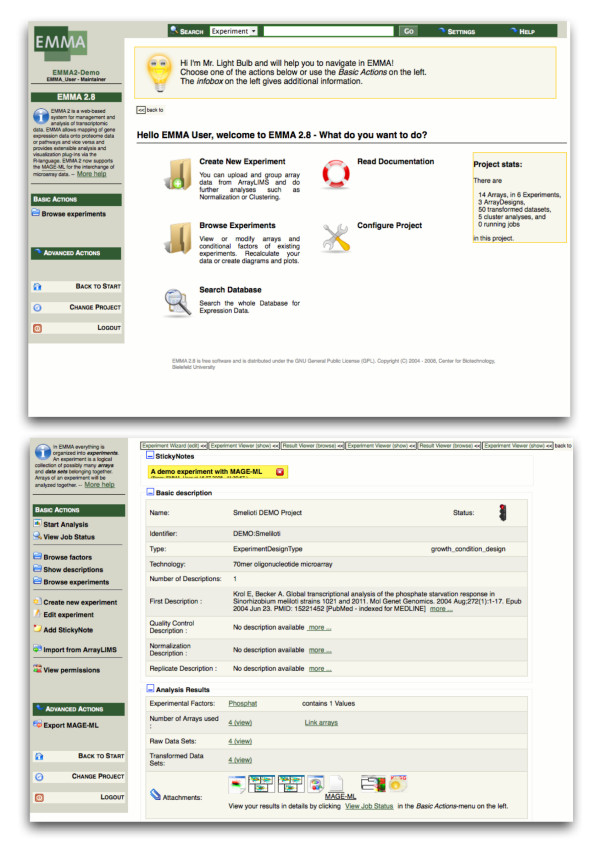
**The web-interface of the EMMA 2 software**. EMMA 2 provides a highly user friendly web-interface with integrated help and documentation. The top image depicts the entry page to each project. The image below shows the overview of an experiment. The outcomes of data-analyses are depicted as icons at the bottom of the page.

**Figure 3:**
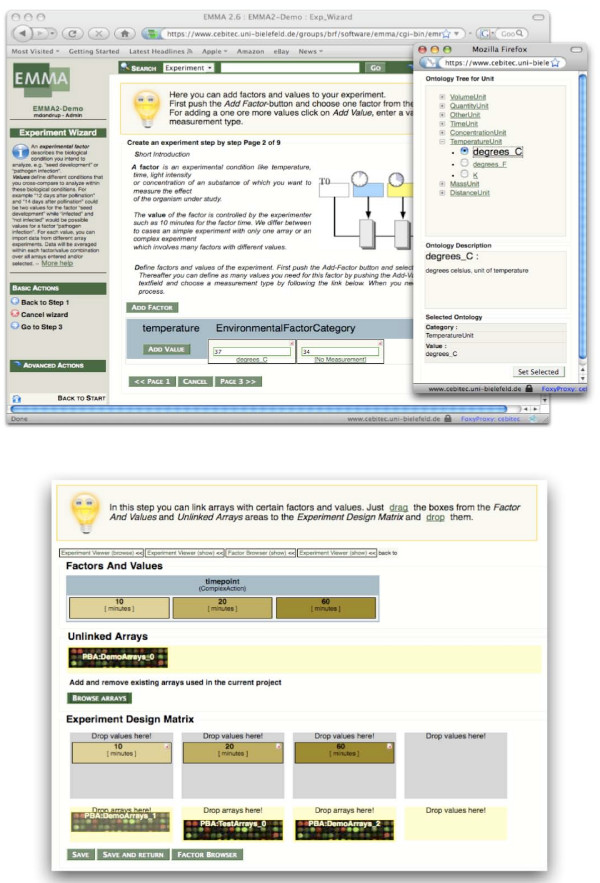
**Management of experimental factors**. Experimental factors and their values are integrated in the web-interface. The screenshots show how measurement units can be defined using the MGED ontology (top). Array data can be assigned to factor values by the use of a drag-and-drop mechanism to build the experiment design matrix.

The EMMA 2 software has been developed as a web-application requiring a standards compliant web-browser on the client side. The interface has been designed to be highly user-friendly and interactive. It aids the user in MIAME compliant annotation of microarray experiments while hiding the complexity of the underlying object model. The interface is built around the central notion of an experiment, which forms collections of data and annotation. Further analysis results, such as images or PDF documents, are depicted as attachments within an experiment (Figure 2). For a precise annotation, experimental conditions need to be defined using a controlled vocabulary. For this purpose, EMMA provides an ontology browser containing the MGED-Ontology (Figure 3).

Different levels of context dependent help and documentation are available ranging from usage hints to a detailed user's manual and a documentation Wiki. The interface makes use of modern web-technologies such as Ajax to increase interactivity and responsivenes of the application.

### Analysis Pipelines

The data analysis mechanisms in EMMA 2 have been developed and designed to be both highly flexible and easy to use for users without much experience. Data analysis in EMMA 2 is carried out within so called pipelines. Analysis pipelines are composed of three components represented in the database model: Firstly, *functions *implement the actual R functions used for data analysis. They have input and output data types and a set of parameters. Secondly, *tools *form linear compositions of type compatible functions together with actual values for the parameters. Lastly, *jobs *encompass the combination of tools with data sets of matching input type. Jobs are executed on a grid-computing system.

*Pipelines *represent a linear arrangement of single analysis functions and a set of corresponding parameter settings. All elements of the analysis system are recorded within the database. Pipelines can be pre-defined by project administrators together with appropriate parameter settings. Functions are typed with respect to MAGE-OM classes.

As analysis functions can be arranged in various sequences, a type system is crucial to enforce sensible combinations of analysis functions. The type system has been designed to automatically feed the correct type of data (e.g. measured or derived) into the analysis pipeline. New functions can be easily added to a project by importing an XML document that contains a description of functions and data types using the TOOLS-ML document language designed to closely resemble MAGE-ML. Analysis pipelines can be pre-configured with sensible default values, while more experienced users are able to adjust analysis parameters and choose different input datasets. All pipeline executions, parameter settings, and the resulting data are automatically recorded in the database.

### Normalization and Pre-processing Functions

Pre-processing of raw data is necessary as a first step in a microarray analysis pipeline after image analysis to remove weak or flagged measurements, handle background estimates, and do further transformations. Normalization is commonly used to remove systematic bias from the data and to make empirical distributions between arrays comparable [23].

EMMA 2 provides normalization and preprocessing pipelines for two-color arrays as well as for single channel arrays (e.g. Affymetrix). For two-color arrays we provide median based and intensity dependent normalization (e.g. lowess and print-tip dependent lowess [24]), which are available in the marray-package [25].

For Affymetrix arrays we provide an interface to pre-processing functions available in the affy package [26] such as MAS5, RMA, and MBEI by means of the 'expresso' function and also GCRMA by using the respective package [27]. For quality control, EMMA 2 allows to create PDF documents with various statistics and plots as provided by the R-package AffyQCReport.

For NimbleGen arrays we have implemented the 'Nixpresso' R function in EMMA 2 to adapt RMA backgroud correction, normalization, and summary statistics for single channel analysis. Array descriptions can be uploaded using an ADF or MAGE-ML file.

### Statistical Inference

The second step of a data analysis pipeline is often the identification of significantly expressed genes. Many statistical inference methods have been proposed recently, some of which are applicable for two-sample comparisons and others are also suitable for multi-factorial experiment designs. The range of inference statistics available in EMMA 2 includes one-sample and two-sample t-tests, Wilcoxon's rank-sum statistic, Significance Analysis of Microarrays (SAM) [28], the CyberT method [29], the LIMMA package [30], and ANOVA for multi-factorial designs.

### Cluster Analysis

**Figure 4:**
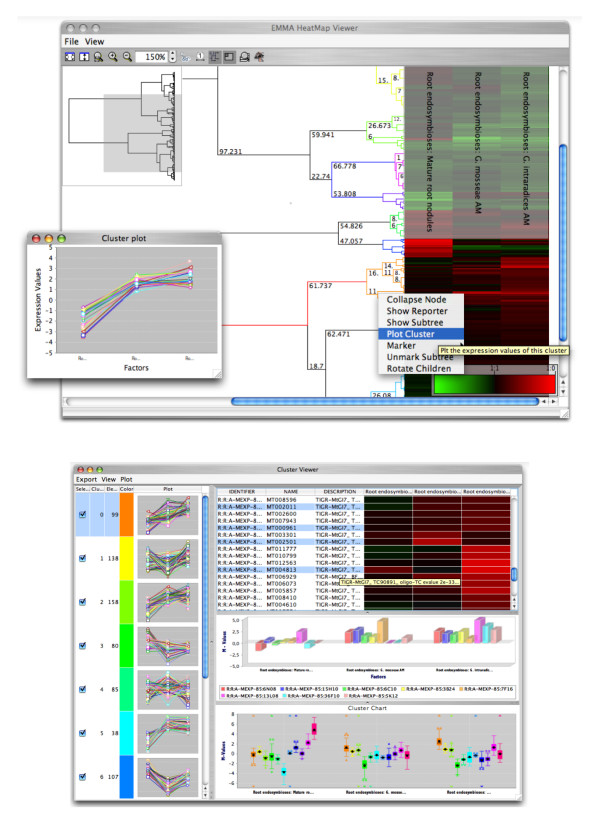
**The integrated applet for viewing clusters**. An integrated solution for cluster analysis is provided. The tree-viewer applet allows intuitive inspection of trees and heatmaps. The tree can be cut at arbitrary heights and the resulting clusters can be further inspected (bottom). Vector and pixel graphics of the tree and the clusters can be exported.

EMMA 2 provides many clustering algorithms, such as agglomerative and divisive hierarchical clustering. All linkage methods provided by R are available by default (Ward's method, average, complete, MacQuitty and Centroid linkage). An experiment can contain an arbitrary amount of cluster analyses to compare different methods and filter settings. The results can be exported in a Newick file or browsed directly with the interactive HeatMap Viewer Java applet (see Figure 4). The HeatMap Viewer allows to cut and inspect the cluster dendrogram and to export dendrograms and heatmaps as vector graphics. The HeatMap Viewer links to the sequence annotation in EMMA.

### Data Integration and Advanced Visualization Features

In EMMA 2, every sequence information (e.g. a reporter oligomere) on a microarray can be crosslinked to the corresponding sequence in the genome annotation system GenDB [31] and vice-versa. The implementation relies on the BRIDGE layer which provides transparent access to the Perl API of remote applications [32].

By communication over this bi-directional layer, functional annotations such as KEGG pathway information and functional classifications are directly accessible. The KEGG-pathway projection is a versatile visualization utilizing the BRIDGE layer. It allows the projection of expression values on corresponding enzymes in a KEGG-pathway graph. Another example application of data integration is the KEGG-violin plot. This novel visualization method allows to depict gene-expression over all pathways. Each violin in the plot represents a KEGG pathway map. The shape of the violin is determined by a smoothed density estimate of gene expression values. Thereby, it is easy to spot pathways which react homogenously to experimental stimuli, and others which exhibit alternate ways of response. Outliers are also depicted within the graphic to easily spot genes which deviate from the general reaction of the encompassing pathways.

Array designs in EMMA can also link to annotation data from remote databases by means of web-services. Web-services present a popular standardized mechanism for data-interchange via internet protocols independent of the programming language. EMMA 2 supports the SOAP [33] messaging protocol within its communication layer to implement web-service support. Communication between EMMA 2 and other applications is bi-directional, while EMMA 2 serves as a web-services provider that enables querying expression data using a given sequence identifier.

The opposite direction of communication is represented by the web-services client built into the communication layer. The client allows to connect sequence data to external web-services in a similar way as it allows to add links to external database resources. All web-services that use the Web Services Definition Language (WSDL) can be directly accessed without further programming. WSDL is an open standard to describe web-services. The web-services connected to a sequence can be queried whenever a sequence is viewed. The results of such queries can be added to data sheets from statistical tests. In particular, regulatory networks can be retrieved from the CoryneRegNet software [34]. CoryneRegNet is a data-warehouse of regulatory interactions for *Corynebacteria*. A reference implementation of such an integrated application framework between transcriptomics data (EMMA 2), a genome annotation system (GenDB) and CoryneRegNet is provided by the CoryneCenter system [10].

### Collaborative Functions

Collaborative functionality is an essential requirement in large distributed projects involving many individuals and institutions. In such projects it is crucial for research groups to easily share experimental data and jointly conduct the annotation of biological experiments and analyze microarray data. Moreover, after a certain level of quality is reached, an experiment needs to be published within the consortium. Furthermore, control on who can or cannot view, export, or edit data is required.

#### Authentication and Authorization

Authentication by username and password is handled by the General Project Management System (GPMS) that is used for all CeBiTec software. GPMS supports the management of multiple projects and provides a role-based authorization model. EMMA 2 comprises of a collection of roles suitable for different degrees of user experience or trust from system administrators who can perform each possible action to an less privileged guest user. Users and accounts can be managed via the GPMS web-interface.

#### Access Control and Shared Data

Although role-based access control is already very flexible, it is not suitable to model access rights to individual objects, as objects are dynamically created and destroyed during run-time. Therefore, we have implemented another layer of control based on Access Control Lists (ACL). ACLs can be used to bind access privileges of users or groups of users to certain objects within the database.

In the repository, each MAGE-OM object can have its own set of ACLs to grant or restrict access to it. Initially, the user creating an object is recorded as its owner and can change access privileges by adding or removing ACLs to the object. ACLs work intuitively for users and groups of users, such that for example granting the 'view' right on an Experiment to the predefined group 'ALL' will make the Experiment visible to all members of a project.

### Applications

EMMA 2.0 became available in 2004 and has been constantly developed and maintained since then. The current version 2.8 is applied in various national and international functional genomics projects.

EMMA 2.8 is the central transcriptomics server for the GenoMik-Plus initiative. GenoMik-Plus is a project funded by the German Federal Ministry of Education and Research (BMBF) to foster genomic research in microorganisms. Within the network EMMA 2 has been used to characterize the gene expression of the model organisms *Sinorhizobium meliloti *[35,36], *Corynebacteria *[37-39] and *Xanthomonades *[40] under many different growth conditions. EMMA 2 is also the central microarray analysis platform of the plant-genomics projects MolMyk [41] and Grain Legumes [42,43] as well as for the EU Network of Excellence Marine Genomics. Altogether, the EMMA 2 databases contain microarray data of more than 20 different organisms. In total, over 3000 microarrays provided by the microarray core facility at the CeBiTec or external project partners and commercial vendors have been processed using the system.

### Application Studies

To demonstrate the capabilities of the system, we have collected data from studies which have used EMMA 2 for microarray analyses within a demo project. These studies are related to the reconstruction of regulatory networks in bacteria and the study of beneficial plant-microbe interactions.

#### Genome-wide Reconstruction of Gene Regulatory Networks in Bacteria

Global transcription profiling with DNA microarrays is an essential prerequisite for the experimental reconstruction of microbial transcriptional regulatory networks. In particular, the detection of differential gene expression upon environmental changes or in defined regulatory mutants provides *in vivo *data on transcriptional control circuits and the contributing gene regulatory networks. The EMMA 2 platform, in conjunction with a whole-genome DNA microarray produced on glas slides, was used for the systematic analysis of expression changes in gene regulatory mutants of *Corynebacterium glutamicum *[44-46].

Differential expression patterns provided the seed information to search for common transcription factor binding sites in the upstream region of the detected genes by pattern matching approaches. For instance, the deletion of the regulatory gene *ltbR*, supposed to be involved in leucine-dependent gene regulation of *C. glutamicum*, revealed surprising results, since the evaluation of the microarray data with EMMA 2 showed differential expression not only for genes involved in leucine biosynthesis, but also for the tryptophan operon. In general, these data can be evaluated *in vivo *by real-time reverse transcription PCR or *in vitro *by electrophoretic mobility shift assays. Accordingly, EMMA 2 substantially supported the reconstruction of regulatory interactions in *C. glutamicum *on a global scale [47].

#### Gene Expression Analysis of Plant-Microbe Interaction

Legume plants establish two different endosymbioses with soil microorganisms: the nitrogen-fixing root nodule symbiosis and the arbuscular mycorrhiza (AM). Whereas nodulation is almost exclusively restricted to legumes and requires the organogenesis of a root nodule that houses the rhizobial prokaryotes capable of symbiotic nitrogen fixation, more than 80% of terrestrial plants enter an AM with fungi of the phylum *Glomeromycota *[48].

In a recent study, we applied 70 mer oligonucleotide microarrays (Mt16kOLI1) representing app. 35% of the gene space of the model legume *Medicago truncatula *to specifiy the overlapping genetic program activated by two commonly studied microsymbionts, *Glomus mosseae *and *Glomus intraradices *[49]. In total, 201 plant genes were significantly co-induced at least 2-fold in either interaction. A range of well-known AM marker genes, e.g. the gene encoding the phosphate transporter MtPt4, were found to be activated, thus validating the transcriptome data obtained. Using the same microarrays, we have studied the transcriptome of nitrogen-fixing root nodules induced by *Sinorhizobium meliloti*. Comparisons of the data sets obtained and cluster analyses revealed the co-induction of only a limited number of genes during both nodulation and mycorrhization. Amongst those were gene functions associated with later stages of the symbiotic interaction, e.g. encoding proteins associated with the specific modification of plant membranes that enclose the symbiotic microbes [50]. The comparative analysis of the symbiotic transcriptome is facilitated by the fact that the reporter sequences stored in EMMA 2 are linked to the SAMS software [51] that provides automated annotations, functional classifications, and the possibility to map expression data onto metabolic pathways. We are currently extending our analyses by using the more genome-wide Affymetrix Medicago GeneChips^® ^for expression profiling. Similar to the EMMA 2 analysis platform for 70 mer oligonucleotide microarrays, a link to the SAMS software allows to connect probe annotations and expression information. In summary, the use of the EMMA software platform has substantially facilitated research on the transcriptome of legume root endosymbioses [41].

#### Performance

The performance of a database system greatly depends on the hardware and software infrastructure as well as on an efficient database implementation. Although the system can be installed on a single LINUX box in principle, we decided to integrate it into our server architecture using specialized shared application servers for performance reasons. The current reference installation of EMMA 2 runs on a SunFire V880 (8 Sparc CPUs, 32 GByte RAM) application server. Two redundant SunFire X4600 servers with attached storage (Fibre Channel, FC-AL) are used as database servers running MySQL 5. Computational jobs are executed on a cluster of dual-core opteron CPUs (Sun v20z, up to 2.8 GHz, up to 32 GByte RAM). All servers are linked to our network via gigabit ethernet. All servers are running Solaris 10 and are shared between multiple software applications.

With this set-up, the import of the array design of the Mt16kOLI1 microarray (≈ 32.000 features, 16.000 reporters) from a generated MAGE-ML file takes approximately three hours. A MAGE-ML export of an experiment containing six Mt16kOLI1 arrays takes approximately 4 minutes without including the ArrayDesign package and 60 minutes with the ArrayDesign included. The performance of general analysis jobs such as normalization and clustering depend on the respective implementations in R.

All further database-based operations, such as searching for expression data for certain genes are executed in a timeframe of seconds. All file-based operations on large data from HDF5 files such as sorting and filtering are executed within milliseconds.

## Discussion

We have implemented a web-based system for the integrated storage and analysis of microarray data which incorporates all major microarray formats. It supports the whole MAGE-OM and provides an object-oriented API to its underlying database model. It is built using a flexible object-relational mapping approach which allows for a high level of automated code generation during the development process. Despite the complexity of the underlying data model, EMMA 2 provides a user friendly web-interface that hides the complexity of the model. The current level of usability is achieved by using simplified metaphors and modern web technologies throughout the interface.

MAGE-OM is a data model that is known for its high complexity and was originally designed as a document format. We had to evaluate MAGE-OM to see if it satisfied the requirements of a data storage model for real-world software applications. There was a significant need to increase the efficiency of the derived data model with respect to storage requirements and query speed. Despite the complexity of the model, the implementation in EMMA 2 demonstrates that with minor extensions and by the use of a few manual optimizations MAGE-OM provides high flexibility and efficiency in the targeted domain.

With respect to system performance we could demonstrate that database indices and database views are highly important optimization techniques as well as file based storage mechanisms for large matrices. However, in order to improve database efficiency for Affymetrix arrays, we restrict our treatment of array designs to CompositeSequences and do not model each individual probe within our database because this would result in the creation of 2 to 10 million database objects, which are never used in subsequent analyses.

In comparison to the previous data model of the EMMA 1 software the increase in flexibility and complexity is evident. The EMMA 1 model consists of approximately 20 classes without inheritance, while the full EMMA 2 model consists of over 200 classes with deep inheritance relations imposed by MAGE-OM. The use of a model driven approach has been crucial for the implementation of such a software project, even though it is not used very frequently in current bioinformatics applications. It might turn out to become highly relevant for the design of other repositories or general database applications in this field in the near future. The main reason we see for this development is the need for novel software applications following the rapidly evolving trends in high-throughput methods and the resulting need for new open standards to annotate results.

The implementation and integration of an ontology browser in EMMA 2 allows for a structured annotation and retrieval of data derived under certain experimental conditions. The integration of external data sources offers the potential to generate novel hypotheses, where results relying on expression values alone would not be sufficient. The system consists of reusable parts such as the Perl API for MAGE-OM, the pipeline system, and the access control model, which could also be beneficial for other database applications. Unlike the MAGE Software Toolkit (MAGE-stk [52]), which provides code-generators for creating document APIs to MAGE-ML, the EMMA 2 API provides functions for storage and retrieval of MAGE data in a database interface. Our implementation provides additional MAGE importers and exporters and reaches an increased flexibility over MAGE-stk in this repsect.

Cluster analysis is a commonly used step in the analysis of gene expression data. Most integrated systems (for example MARS and BASE) do not provide an integrated tool to compute and view cluster analyses within the system. ArrayExpress provides ExpressionProfiler as an integrated solution to cluster analysis. Nevertheless, the process of selecting the correct data columns from a large data matrix is rather complicated and the results cannot be stored within the database. In EMMA 2, the clustering process has been simplified by using pipelines, as they allow us to feed compatible data types into the analysis automatically.

The ability of the EMMA 2 software package to integrate data from external data sources via two different communication channels is an outstanding feature. The level of bi-directional web-service integration is unique when compared to other microarray analysis systems and also in comparison to public repositories such as ArrayExpress, which of course have a different focus than EMMA 2. ArrayExpress provides programatic access via web-services to raw experimental data and also access to raw and processed data via its web-interface and provides some data analysis tools. In addition, EMMA 2 also allows to query normalized data via its SOAP interface.

The flexibility of its analysis pipelines is another important advantage of the presented software. Especially with respect to high-throughput data it is beneficial to be able to distribute the compute load to a compute cluster. Such a system could also be applicable for novel methods in transcriptomics such as high throughput sequencing techniques. It will be highly interesting for future database applications to integrate array based and sequencing based transcriptomics approaches.

### Outlook

The system is under active development. Support for NimbleGen microarrays is currently in the testing-phase. Furthermore, we are working on integrating quantitative matabolomics data, for example from our novel MeltDB software [53] via web-services. We further intend to develop the system into a general transcriptomics platform by incorporating new pipelines. These could include fast mapping algorithms which will allow to map sequenced tags to the genome sequence efficiently and normalization and statistical test for count data. While the MAGE-OM model could be partially sufficient for the purpose of experimental annotation and quantitative data, building repositories for handling ultra-fast sequencing transcriptome data will require more effort in designing efficient database systems.

## Conclusion

We have developed a versatile system for the integrated analysis of microarray data, called EMMA 2. It is unique in its cummulated support for two-channel and Affymetrix analysis. Adherence to public standards such as MAGE-ML and the MGED ontology is a prerequisite for the reproducibility and interpretability of microarray experiments. The system also supports data integration by use of standard protocols such as web-services that is unprecedented in other software systems. The overall concept of the system could be shown to be adequate and extensible to new technologies. The system has contributed to many collaborative functional genomics studies with many international contributors and is actively used and developed. Due to its flexibility and efficiency, the system seems well suited to be extended for the analysis of quantitative transcriptomics data from the novel ultra-fast sequencing technologies.

## Availability

• The EMMA 2 server and documentation is available at: 

• Demo projects with representative data have been set up.

• Source code packages are available under the GPL upon request.

• System requirements for local installation: UNIX/Linux, Apache, MySQL, Perl, R, and Bioconductor, and additional system libraries, RSPerl.

• Local installation requires skills in Linux/UNIX administration.

• Web-client requirements: Java-enabled web-browser (preferably Mozilla Firefox).

• Instructions on usage of the programmatic SOAP interface are available at: 

## Authors' contributions

MD designed and implemented most of the core system and wrote the manuscript. SA, SJ, JG, FT, and TK contributed to the web-design and implementation. DM implemented and evaluated statistical inference methods. HK and AT provided substantial intellectual contribution to the manuscript and provided the application cases. TK implemented the LIMS component. TG and FT developed Java Applets. KH and CKK built analysis pipelines and parsers for Affymetrix and NimbleGen. BL contributed the O2DBI layer within our model-driven approach. VM contributed to the documentation and manuscript. HN and AG implemented data integration layers and contributed substantial intellectual input to the manuscript. KJR developed the MAGE-ML exporter generator framework. KJR and FT integrated the ontology. AP and AG initiated, supervised, and directed the whole project. All authors read and approved the final manuscript.
